# Kidney organ donation: developing family practice initiatives to reverse inertia

**DOI:** 10.1186/1472-6963-10-127

**Published:** 2010-05-17

**Authors:** Emmanouil K Symvoulakis, Emilia Stavroulaki, Myfanwy Morgan, Roger Jones

**Affiliations:** 1 Department of Blood Donation, University General Hospital of Heraklion, Crete, Greece; 2 King's College London, Department of Primary Care and Public Health Sciences, London, UK

## Abstract

**Background:**

Kidney transplantation is associated with greater long term survival rates and improved quality of life compared with dialysis. Continuous growth in the number of patients with kidney failure has not been matched by an increase in the availability of kidneys for transplantation. This leads to long waiting lists, higher treatment costs and negative health outcomes.

**Discussion:**

Misunderstandings, public uncertainty and issues of trust in the medical system, that limit willingness to be registered as a potential donor, could be addressed by community dissemination of information and new family practice initiatives that respond to individuals' personal beliefs and concerns regarding organ donation and transplantation.

**Summary:**

Tackling both personal and public inertia on organ donation is important for any community oriented kidney donation campaign.

## Background

Kidney transplantation is the preferred treatment option for end stage renal failure in terms of mortality [1,2], cost effectiveness [3], and improvement in quality of life [4]. The average dialysis treatment cost per life year saved is estimated at approximately $55,000-80,000 [3], while for kidney transplantation it is estimated at approximately $10,000 per life year saved [3]. Increased time on dialysis is related to higher rates of kidney failure twelve months after transplantation [5], and to decreased survival of transplant recipients [6,7].

During the period 1995 and 2004 in the United States, the number of patients on the transplant waiting list has increased by 79% [8]. The number of new registrations continues to grow over time. Not surprisingly, 22% of waiting list patients in 2004 had been waiting for 3 years or more, compared with a 14% in 1995 [8]. In the UK, there was a 16% increase in the number of patients on the kidney or kidney and pancreas transplant list between 2005 and 2008 [9]. In Greece, the number of patients on the kidney transplant list increased by 12% over the same period (2005-2008) [10]. The increasing demand for organ transplantation has led to a growing interest in living organ donation, which now makes up approximately half of all kidney transplants in the USA [8,11]. In the UK, the annual deceased donor kidney transplant rate was 23.5 and in Greece 9.2 per million population (pmp) respectively, during 2007. In the same year, the living kidney transplant rate was 13.4 and 7.9 pmp for the UK and Greece respectively [12]. Data from various regions suggest that more than half of the countries surveyed reported at least a 50% increase of the number of living kidney transplants in a ten year period [13]. Most living donors (67%) are genetically related to the recipient, but there has been a 10-fold increase in the number of transplants from unrelated donors in the last ten years [11,14]. This tendency to accept more unrelated donors is largely due to the finding that graft and patient survival rates are comparable between living related and living unrelated donor transplantation [14,15]. The mortality rate of donor nephrectomy has been estimated at 0.03% and of major complications 0.2%, risk rates that need to be considered against the preoperative healthy status of a living donor [16]. However, in some countries with limited institutional and financial resources, issues involving organ allocation process may lead to perplexity and uncertainty in terms of equity among recipient candidates, donor safety and transparency about prioritisation within the waiting list [17]. Cases related to organ removal from non-related living donors and lack of bio-ethical considerations amounting to a 'commercial traffic of organs' have been reported to occur in countries without well-regulated organ donation and transplantation systems. Reports or rumours that organ transplantation may be influenced by the economic status of the waiting list patients have led to negative and suspicious public reactions [18].

The aim of this article is to discuss the complexity of kidney donation issues, by briefly reporting on bio-ethical, organizational or social aspects of donation, and to highlight the need for synchronised community-oriented changes to reverse inertia or negativism to donation, through the pathways of family practice driven services and research initiatives.

## Discussion

### Consent for organ donation

Deceased donation is the ideal treatment option because it improves health status and quality of life for the recipient without interfering with the well-being of a living donor. *Presumed consent *is a donation model where people are presumed to have given consent for donation unless they have previously declared otherwise. Countries such as Austria, Belgium and Spain have adopted this policy, which has been associated with significant increases in deceased organ supply [19,20]. However, some argue that presumed consent is at odds with the principle of decisional and individual autonomy [21]. In the UK, the evidence shows that even those who theoretically support a system of opt out have some reservations in terms of the human rights implications [22]. The infrastructure, information processing and staff training requirements to support this policy model are also considerable [22].

In several countries (including UK and Greece), *informed consent *systems allow the removal of organs from persons who had previously decided to opt in, or whose relatives give consent at the time of death [22]. This opt in model is associated with a greater need for community campaigns to provide information and overcome inertia and negative attitudes to organ donation.

### Culture, ethnicity and donation

In the UK, people of African, Black Caribbean and South Asian heritage are more likely to need a kidney transplant in comparison with the White population [23]. This increased demand for organ donation reflects their higher rates of disorders such as diabetes and hypertension, which are major causes of end stage renal failure [23]. However, organ donation rates among ethnic minorities in the UK continue to be lower in comparison with the donation rates among the White people [24]. Recent data from the UK Transplant Activity Report 2008-2009, show that 94.9% (554/584) of deceased heart beating kidney donors were White [9]. Only 2.1% (12/584) and 1.2% (7/584) respectively were Asian and Black donors [9]. Asian and Black groups make up 22.7% (1629/7190 patients) of the kidney waiting list and represented 19% (208/1096) of the transplant recipients in 2008-2009 [9]. Several studies identify this as reflecting differences in knowledge about transplantation and more negative attitudes to donation [25,26]. Concerns about the need for the body to remain intact at burial have been reported [25,26]. Morgan *et al*. suggested that people of Caribbean heritage living in a multi-ethnic area of south London believe that keeping the body intact to be returned to the land of origin represents a way of reconciling at death their experience of a divided identity and sense of dislocation as a member of a minority ethnic group [25]. The effects of deprivation and feelings of marginalization may also be associated with mistrust in doctors and the medical system, including concerns that organs might be used for medical research or that less effort will be made to save the lives of potential donors. These concerns, together with worries about 'tempting fate' by carrying a donor card, may also lead to negative attitudes or inertia in registering as kidney donors [26]. Reluctance among some groups to donate organs outside their 'narrow' community may also enhance the disconnection between the need for organs and their availability [26]. Additionally, religious beliefs and practices regarding appropriate handling of the body have been identified as contributing to uncertainties and negative attitudes to organ donation among some minority groups. This occurs despite declarations made by leaders from all the major faith groups in the UK that there are no religious prohibitions against organ donation [27,28].

### Organ donation in Greece

Organ donation is a complex area in terms of bio-ethical, organizational, financial and social barriers. It is difficult to provide a full explanation for negative, neutral or ambivalent perceptions. In 2006, only 3% of Greek citizens carried an organ donation card [29]. In our recent study on the island of Crete (Greece), among the 224 primary care users from two rural areas over 61% had concerns that organs might be used for different purposes like medical research [30]. A sizeable minority of respondents (25.6%) were worried that registering as donors is like tempting death, despite the fact that about 95% of the participants did not find organ donation unacceptable because of religious issues [30]. Finally, there was a large gap between the proportion of people actually registered as donors (2.2% of respondents) and those who state that they are willing to register as kidney donors (45.7%) [30]. A similar gap has been noted in other countries, with a key policy objective being to close this gap and achieve a higher donor registration rate by people who are willing to donate [22,31].

Although the major focus of research has been on the variations in attitudes to donation and donation rates among ethnic minorities, we observed that primary care users living in rural and remote mountain areas in Crete have more negative attitudes to donation compared with more urban areas [30]. This observation supports the hypothesis that the socio-demographic characteristics of communities may influence knowledge and attitudes to organ donation, with diversity in beliefs and attitudes extending beyond ethnic groups.

### Information about donation

Geographical barriers may create clusters of groups, holding rigid traditional values and cultural practices [25,30]. On the other hand, in Greece, increases in new donor registrations following media publicity about successful cases of transplantation are usually transient [32]. These observations lead to the idea that interventions should be community-oriented in order to improve people's attitudes to donation and to avoid risks of adverse reactions when treating fragile personal, family and social values. We believe that primary care could play a greater role in educating patients about organ donation [33-35]. The implementation of educational programmes by primary health care professionals about organ donation and transplantation could favourably and rapidly influence the attitudes and knowledge of potential donors. In the USA, results from a prospective randomized study support the belief that family physicians can increase the commitment of their patients to organ donation [36]. A statistical significant difference was found between pretest and posttest scores assessing knowledge regarding organ donation [36]. Furthermore, an additional positive effect reported was the recruitment of family members of the patients as new donors [36]. In the USA, the findings of a cross-sectional mail and Internet-based survey showed that only 4% of physicians had discussed organ donation with their patients and 11% had relevant information in their practice [37]. Most had a limited knowledge about organ donation [37] and two thirds of the physicians (64%) said that they did not sufficient staff to adequately approach the issue of donation [37].

Critical care units and their specialised staff play a crucial role in families consent to donation, with higher consent rates influenced by the duration of the consent discussion and responses to families' worries and concerns [38]. However, communication within family practice has also been shown to influence attitudes and practices and increase donation rates by preparing people to think about organ donation well in advance of an emergency hospital admission [39]. Such communication requires taking into account human, cultural and social diversity [40,41] to overcome interpersonal barriers and open up dialogue with the public [30]. Approaching bio-ethical issues in a simple but transparent manner may allow effective dissemination of messages between primary care providers and users and encourage informed choices about kidney donation [42]. Discussion of donation with one member of a family may also have an amplifying effect by triggering discussion and shaping the attitudes of other family members [43]. It has been reported that school based educational courses or group meetings chaired by people with experience of a specific disease or health problems, are likely to be effective in shaping personal views [44]. It has also been shown that short lecture sessions can impact on personal intentions towards organ donation among adolescents and young students [45,46].

Similar youth-oriented pathways and support group educational activities could be explored within family practice. Dissemination of information concerning kidney organ donation among younger members of the families and adolescents may be effective in changing personal and family attitudes towards donation [41].

It is important to highlight the role of the general practitioner [47] and the need to involve other health professionals [48], including nurses, social workers and psychologists working in primary care, to approach issues of kidney donation, donor recruitment and commitment in ways that take account of social and cultural beliefs. An integrated primary health care team in conjunction with a specialist or transplant expert trained to educate and to gain knowledge from the prospective donor has been shown to provide informed decision-making and the 'perceived integrity of living organ donation' [48]. Group meetings among primary care users and staff can allow discussion of complex issues such as living unrelated donation and such interaction is likely to offer new insights. Consultations in primary care settings may help, both to recruit people to register as donors, and allow the testing of different approaches including short opportunistic verbal interventions during the ordinary visits to reduce uncertainties and limitations of medical, legal and ethical information [49]. Finally, incentives provided to the involved health care professionals, could be linked to improvements in the annual donor registration rate in each primary care district. For example, developing performance indicators in family practice based on the annual number of primary care users who are newly registered as donors may deserve further discussion.

The role of research within family practice is important in order to examine specific issues of concern and to target knowledge dissemination for the benefit of the whole community. Recently, the conclusions of our rural primary care study [30] have been welcome by a major editorial in a national newspaper [50]. The need for knowledge transfer and for joint interventions in order to improve counter negative attitudes and rates of organ donation, by simply thinking that anybody could find themselves a potential transplant recipient [50], emerged and was fed back as amplified messages targeted at the wider readership. This was the first study aiming to describe current opinion regarding kidney donation among primary health care users in rural settings in Greece [30]. As a next step, it will be important to continue to collect data from sub-groups with different socio-demographic backgrounds in order to design better-focused community campaigns and evidence driven educational activities.

## Summary

Ongoing growth in the number of patients with kidney failure and limited availability of kidney transplants leads to long waiting lists and poor quality of life. Negative, neutral or ambivalent organ donation perceptions often interlace between bio-ethical, organizational and social limitations. The implementation of family practice driven information and education campaigns about organ donation and transplantation has the potential to increase the numbers of new donors. The role of research within family practice is important in order to disseminate knowledge regarding organ donation.

## Competing interests

The authors declare that they have no competing interests.

## Authors' contributions

EKS had the original idea for the paper which was written and revised by all authors.

## Pre-publication history

The pre-publication history for this paper can be accessed here:

http://www.biomedcentral.com/1472-6963/10/127/prepub
